# Breaking the habit: a systematic review of the cost-effectiveness of non-pharmacological and combined interventions for smoking cessation in Europe

**DOI:** 10.1007/s10198-025-01855-7

**Published:** 2025-10-29

**Authors:** Alexander Braun, Walter Hyll, Eva Krczal

**Affiliations:** 1 Centre for Evidence-based Health Services Research, University of Continuing Education Krems, Krems, Austria; 2 Institute for Management and Economics in Health Care. UMIT TIROL, Private University for Health Sciences and Health Technology, Hall in Tirol, Austria; 3 Department for Economy and Health, University of Continuing Education Krems, Krems, Austria

**Keywords:** Smoking cessation, Cost-effectiveness analysis, Systematic review, Europe, I18

## Abstract

**Background:**

Smoking is one of the main causes of preventable disease and premature death. While existing evidence syntheses the cost-effectiveness of smoking cessation interventions for hospitalized patients and high-risk groups such as pregnant women or children, the evidence on the cost-effectiveness of non-pharmacological interventions for the general population remains relatively underdeveloped.

**Methods:**

A systematic literature review was performed using MEDLINE, EMBASE, Cochrane Library, and NHS Health Economic Evaluation Database along with grey literature, pre-prints, and HTA reports.

**Results:**

A total of 9,541 abstracts were screened, with 23 studies meeting the eligibility criteria. These studies focused on four main intervention types (i) face-to-face counseling, (ii) digital/telephone counseling, (iii) reimbursement, and (iv) awareness building. The ICERs ranged from − 332,320 EUR/QALY to 156,310 EUR/QALY. Of the 39 ICERs reported, 30 (76.9%) demonstrated superior cost-effectiveness for smoking cessation interventions. Nine studies reported strong dominance, where the intervention not only gained QALYs but also saved costs. On average, 0.02 QALYs (SD = 0.02) were gained per person. When Life-Years Saved (LYS) were used as the effectiveness measure with a range from EUR 192/LYS to EUR 17,908/LYS. All ICERs werebelow the EUR 25,000/LYS willingness to pay threshold.

**Conclusion:**

The evidence suggests that smoking cessation interventions are general cost-effective in Europe. Personal counseling appears crucial for digital interventions to demonstrate cost-effectiveness. Also, awareness building could serve as a cost-effective means of supporting existing cessation programs.

**Supplementary Information:**

The online version contains supplementary material available at 10.1007/s10198-025-01855-7.

## Background

Tobacco use represents one of the leading preventable health risks, causing over 8 million deaths globally each year [1, 2]. Despite a declining trend in smoking prevalence since the 1990s, Europe still records high smoking rates with approximately 18% of smokers, particularly in Central European countries which show the highest smoking-related mortality [3]. The health consequences of tobacco use, including malignant neoplasms, cardiovascular and obstructive respiratory diseases are also well established [4], as are the risks of passive smoking, such as sudden infant death syndrome, asthma, and cardiovascular disease [5, 6]. Smoking-related diseases also impose a substantial economic burden in most Western European countries [7]. Health economic analyses, considering productivity loss and health expenditures, have shown that smoking-attributable diseases account for around 2.5% of the regional annual Gross Domestic Product [8]. In 2024, the total cost of smoking-related diseases was estimated at EUR 692.8 billion, with a labor productivity loss equivalent to 7.1 million years due to disability and mortality [8]. Health economic evaluations of smoking cessation programs provide valuable data to appraise the effectiveness of interventions and support optimal resource allocation in healthcare [9]. Several literature reviews have demonstrated the cost-effectiveness of such programs, particularly in specific subgroups including hospitalized patients [10], adolescents [11], individuals with chronic diseases or pregnant women [5, 12] Considering the type of intervention, existing reviews largely focus on pharmacological interventions to assess cost-effectiveness [13–15].

But there are several limitations to the current evidence: (1) There is a focus on people with specific pre-conditions, such as hospitalized patients, chronically ill populations, or pregnant women, as well as evaluations addressing preoperative smoking cessation or relapse prevention [5, 10, 12, 16–18]. (2) There is limited evidence on the cost-effectiveness of smoking cessation programs aimed at the general population as a primary prevention measure. (3) Existing reviews predominantly evaluate pharmacotherapies, comparing the cost-effectiveness of various nicotine-free medications and nicotine substitutes or evaluating pharmacotherapies against behavioral interventions [9, 13–15, 19]. Additionally, a review from 2009 highlighted methodological shortcomings in the quality of CEA studies, though the volume of published studies has since increased [20]. Considering the high economic burden of smoking in Europe, this systematic literature review aims to provide evidence on the comparative cost-effectiveness of behavioral smoking cessation programs targeting healthy individuals in Europe. We seek to address the existing research gap by focusing on behavioral interventions for the general population as a form of primary prevention. This review contributes to the current health economic evaluation literature by synthesizing evidence and comparing the results of cost-effectiveness analyses.

The present systematic review contributes to the current literature two aspects. Firstly, it demonstrates that behavioural programmes for smoking cessation are cost-effective to a broad extent, irrespective of whether they take place face-to-face or virtually. Secondly, we demonstrate the existence of evidence that would indicate the inclusion of avoided productivity loss would result in the potential for cost savings of smoking cessation for society. The remainder of this paper is structured as follows: First, we explain the methodology of the systematic literature review and the search strategy used. Next, we present the criteria for assessing eligibility. This is followed by a presentation of the search results and a narrative description of the studies analyzed. We then detail the cost and effectiveness parameters for the four identified categories and discuss the incremental cost-effectiveness ratio of the studies. Finally, we synthesize the evidence across all studies, compare it with existing literature in a discussion, and summarize the limitations and conclusions of the review.

## Methods

We applied a systematic literature review approach, following the guidelines of the Preferred Reporting Items for Systematic Reviews and Meta-analysis (PRISMA) and the recommendation of the Centre for Reviews and Dissemination (CRD) of the University of York [21, 22]. The PRISMA checklist can be found in the appendix (A1). The search was performed on June 18, 2024, using MEDLINE (via PubMed), CINAHL (via EBSCOhost), the Cochrane Library, and the National Health Service Economic Evaluation Database (NHS EED) (via CRD). All published studies to this date were included for abstract screening. In addition, we searched for preprints, grey literature, and Health Technology Assessment Reports in medRxiv, CADTH Grey Matters, and the International HTA database between June 18 and July 31, 2024. The MEDLINE search query included Medical Subject Headings (MeSH), and the keywords were adapted and applied to EMBASE, the Cochrane Library, and the NHS EED. We used the semi-automatic review program COVIDENCE for our review. The systematic review was registered at PROSPERO (CRD42024574630) on August 12, 2024.

### Search criteria

Based on the PICOS (Population, Intervention, Comparator, Outcomes, Study Design) scheme, we developed the search strategy [23] and defined the inclusion and exclusion criteria (Table 1).

**Table 1:** Inclusion and exclusion criteria based on PICOS

	Inclusion	Exclusion
Population	Smokers in all conditions and settings in Europe over the legal smoking ban	• Smokers with specific indications (cancer, COPD, diabetes) • Smokers with specific needs (substance abuse disorders, mental health conditions, etc.) • Population subgroups including pregnant women, families with newborns, specific ethnicities, deprivation levels, certain age groups, etc.
Intervention	Smoking cessation programs including behavioral interventions, digital tools, mass media use, provision of information, financial incentives with a follow-up of at least 6 months	• Studies comparing solely pharmaceutical interventions • Temporary, short-term cessation for medical procedures • Substitution without the goal of abstinence
Comparator	Usual (standard) care, no intervention, placebo intervention	E-cigarettes or vaporizing products as a substitute
Outcomes	Direct & indirect costs; Net-Benefit, Life-Years-Gained (LYG); quality-adjusted life years (QALYs); number of smoking quitters; Incremental Cost-Effectiveness-Ratio (ICER); Incremental Net-Benefit (INB)	Single health outcome without cost assessment
Study design	· Cost-Effectiveness-Analysis, Cost-Utility-Analysis (alongside RCT, based on cohort studies or modeling) · Perspective of public health care funders and/or societal perspective	Patient’s perspective

Abstracts and full texts were screened by two independent reviewers (EK, AB, WH). Any disagreement or uncertainty was discussed and resolved in a meeting with at least three reviewers. The search query can be found in the appendix (A2).

## Analytical framework

The classification of smoking cessation interventions in this review is guided by the transtheoretical model, which conceptualizes behavior change as a process involving six stages: precontemplation, contemplation, preparation, action, maintenance, and termination. The model illustrates how individuals progress from not considering quitting to actively engaging in cessation efforts and, ultimately, sustaining long-term abstinence [24, 25]. In the context of smoking cessation, interventions can be designed to address specific stages of behavior change. For example, preventive approaches such as awareness campaigns draw smokers’ attention to the negative effects of smoking (Precontemplation) and induce the intention to quit (Contemplation). When individuals move into the preparation and action stages, more intensive interventions, such as face-to-face counseling, become relevant to support the initiation of abstinence. Digital tools and telephone counseling can play a crucial role, particularly during the maintenance stage, by providing ongoing support and relapse prevention. Finally, reimbursement schemes can act as contextual enablers, facilitating access to cessation programs and reducing financial barriers, thereby promoting equity in the utilization of these interventions.

Based on these theoretical considerations, we categorized smoking cessation interventions into four groups : (i) face-to-face counseling in groups or one-to-one settings to support smokers in quitting and maintaining abstinence; (ii) digital support and telephone counseling, primarily used for maintenance and relapse prevention; (iii) awareness building to motivate smokers in earlier stages to consider quitting; and (iv) reimbursement, which can serve as a contextual factor to improve access and, if designed accordingly, also as compensation for additional (social) costs of smoking cessation.

## Data extraction

The data was extracted by an experienced health economist (AB) into a pre-defined table, which included the following information: (a) authors, (b) year, (c) country, (d) currency, (e) year of currency report, (f) intervention type, (g) cost type, (h) cost for intervention, (i) standard deviation of cost, (j) sample size of the intervention group, (k) effectiveness of the intervention, (l) standard deviation of effectiveness, (m) control type, (n) costs of control, (o) standard deviation of cost, (p) sample size of control group, (q) effect of the intervention, (r) standard deviation of effect, (s) net-benefit (cost of intervention – cost control), (t) net-outcome (effectiveness of intervention – effectiveness control), (u) reported ICER, (v) discount rate (for costs and outcomes), (w) time horizon, (x) perspective of CEA (societal or healthcare), and (y) type of study design (model-based or RCT). The extracted values were cross-checked by another experienced economist (WH). Furthermore, we calculated ICER and INB for validation if no ICER/INB was reported.^1^ The costs were adjusted to 2023 EUR values using the CCEMG-EPPI Centre Cost Converter (Version 1.7, last update: January 2024), which accounts for changes in purchasing power and the currency conversions based on the values provided by the International Monetary Fund (IMF).

## Quality assessment

Two reviewers (AB and EK) independently evaluated the 23 included studies using the critical appraisal checklist by Drummond et al. [28]. Both reviewers discussed their results in a follow-up meeting to reach an agreement on their appraisal.

## Results

**Fig. 1:**
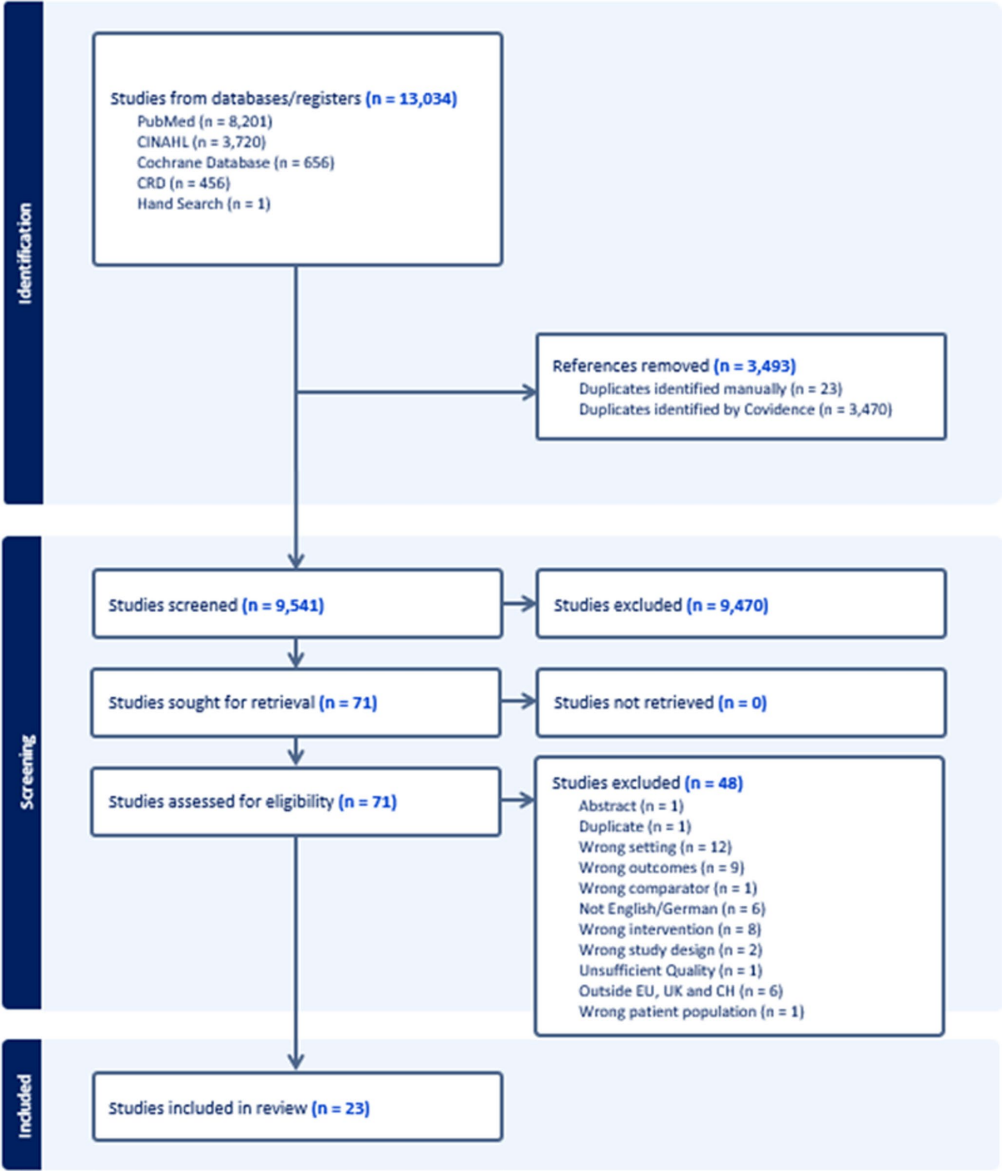
PRISMA flowchart

The literature search identified 13,033 abstracts. 8,201 from MEDLINE, 3,720 from EMBASE, 656 from the Cochrane database, and 456 from the NHS EED (Fig. 1). One study was identified through manual searching. After excluding 3,493 duplicates, 9,541 titles remained for abstract screening. Of these, 9,470 titles were excluded, and 71 full texts were screened for eligibility. Ultimately, 23 studies met the inclusion criteria and were included in the evidence synthesis [29–51].

## Study characteristics

The included evaluation studies are classified into four groups according to the intervention type: (i) face-to-face counseling programs [29, 33, 36, 40, 41, 43, 49], (ii) digital or telephone counseling [32, 42, 44–47], (iii) reimbursement [30, 31, 34, 37, 48], and (iv) interventions for awareness building [35, 38, 39, 51]. A total of six studies were conducted in the Netherlands [32, 37, 41, 44, 45, 48], five studies in Sweden [33, 34, 40, 46, 49], four studies in the United Kingdom (UK) [29, 35, 50, 51], two studies each in France [30, 31] and Spain [36, 47] and one study each in Denmark [42], Germany [43] and Hungary [39]. The studies by Gilbert and Wu refer to a joint study, whereby Wu’s study is an extract from the Gilbert study [35, 51]. We have summarized the results from both studies and assessed their different reporting separately in the evidence synthesis. The publication dates span the entire search period, including studies published between 2004 and 2024. The average population age, reported in eight studies, ranges between 45.7 and 49.3 years [33, 35, 37, 40, 41, 44, 45, 51], while other studies report an average age of under 40 years. The sample size varies and depends on the study design. In clinical studies, the sample ranges between 205 and 6,911 study participants [35, 37, 44, 45, 50]. Modeling studies estimates cohorts between 1,000 and 3.8 million smokers [29–35, 38, 39, 41, 42, 47–50]. Regarding study design, randomized controlled trials (RCTs) and modeling studies predominate. Nine randomized controlled trials [33, 35, 37, 40, 43–45, 49, 50], one non-randomized controlled trial [36], four observational studies [29, 38, 42, 46], and ten modeling studies [30–32, 34, 35, 38, 39, 42, 47, 48] are included. Many studies use the ‘Benefits of Smoking Cessation on Outcomes (BENESCO)’ model, which is considered the standard model for simulating costs and effectiveness from a lifetime perspective. Modeling studies obtain their data from secondary sources. In nine studies (five RCTs and four observational studies), primary data is supplemented by secondary data for long-term projections and integrated into the decision-analytical models. All studies with a time horizon exceeding one year apply a discount rate between 3.0% and 4.0% to their costs. Most studies used the EQ-5D for calculating the QALY [29, 33–36, 39, 44, 45, 47, 49–51]. A total of 69 ICERs are reported in the 23 studies. All descriptives are shown in Table 2.

**Table 2:** Study characteristics: face-to-face counseling

Author, year, country		Study design	Type of analysis	Perspective	Study population, sample size, age, sampling method	Intervention and control	Follow-up duration
Face-to-face counseling							
Bauld, 2011 UK		Observational study, simulation study (Markov)	CEA, CUA	Healthcare	Smokers, NHS SCS participants in Glasgow, having set a quit date *n* = 1,785; follow-up: 22% Markov: 3.000 (Cohort, 1,000 each)	I1: 7-weeks group therapy + pharmacotherapy I2: 12-weeks individual counseling by pharmacist + pharmacotherapy vs. unaided smoking cessation	12 months (cycle)
Feldman, 2019 Sweden		RCT, simulation study (Markov)	CEA, CUA	Society	Smokers between 19–71 years, sufficient language skills *n* = 300, follow-up 84% (1 year), 80% (5–8 years), average age: 49 Recruitment in dental clinic or general practice	High-intensive therapy (counseling in dental clinic) vs. low-intensive therapy (self-help materials)	12 months 5–8 years
Gòmez-Martinez, 2024 Spain		Non-randomized controlled study	CEA, CUA	Healthcare and society	Smokers 18 years or older, average age: 49 Pharmacists assigned to the intervention when having received CAESAR-Training, recruitment by pharmacists *n* = 1078 (I: 278, K: 800) Follow-up: I: 54,32%, K: 60,88%	Behavioral therapy + pharmacotherapy by pharmacists with CESAR training vs. usual care	12 months
Nohlert, 2013 Sweden		RCT, simulation study (Markov)	CEA, CUA	Healthcare and society	Adult smokers, Average age: 49 *n* = 300, follow-up: 86%	High-intensive therapy (counseling in dental clinic) vs. low-intensive therapy (self-help materials) No intervention	12 months (cycle)
Olsen, 2006 Netherlands		Simulation study (Markov)	CEA	Healthcare	Adult smokers, average age: 48.6 *n* = 10,000 (cohort)	SCS offered by hospitals, pharmacies, and other organizations, incl. group- and individual counseling, NRT vs. no intervention	12 months (cycle)
Salize, 2009 Germany		Cluster RCT	CEA	Healthcare	General practice patients between 36–75 years, daily smokers + GP visit for health examination = 577 Recruitment through participating GPs	I1: GP training + GP remuneration for each abstinent patient I2: GP Training + free NRT and/or Bupr I3: Combination I1 + I2 vs. usual care	12 months
Virtanen, 2017 Sweden		Cluster RCT, simulation study (Microsimulation + Markov)	CUA	Healthcare and society	Smokers between 20–75 years *n* = 205 (I: 99, C: 106) Dental clinics assigned to intervention or control	Minimal counseling in dental clinic vs. usual care	6 months
Digital counseling							
Feenstra, 2005 Netherlands	Simulation study (Microsimulation)		CEA, CUA	*Society* (only direct healthcare costs)	Smokers *n* = 1,000 (cohort)	I1: Telephone counseling I2: Minimal counseling I3: Minimal counseling + NRT I4: Intensive counseling + NRT I5: Intensive counseling + Bupr vs. usual care	12 months (cycle)
Rasmussen, 2013 Denmark	Observational study, simulation study (Microsimulation)		CEA	Healthcare	Quitline callers, smokers *n* = 386, follow-up: 76.9% First 100 callers per quarter	Telephone counseling vs. other SCS	12 months (cycle)
Smit, 2013 Netherlands	RCT		CEA, CUA	Healthcare	Smokers, 18 years or older, motivated to quit, sufficient language skills, internet access, average age: 48 years *n* = 414 (I1: 163, I2: 132, K: 119), follow-up: 55,8% Recruitment by Practice Nurse	I1: Web-based and personalized program + one counseling session with Practice Nurse I2: Web-based and personalized program vs. usual care	12 months
Stanczyk, 2014 Netherlands	RCT		CEA, CUA	Society	Daily smokers, 18 years or older, motivated to quit, sufficient language skills, internet access, average age: 45.7 years *n* = 2,099 (I1: 670, I2: 708, K: 721) follow-up: (I1: 54%, I2: 60%, C: 58.5%) Enrollment via the study website	I1: Video-based and personalized program I2: Text-based and personalized program vs. general advise	12 months
Tomson, 2004 Sweden	Observational study		CEA	Healthcare	Smokers, Quitline callers *n* = 1.131	Telephone counseling via Quitline	12 months
Trapero-Bertran, 2018 Spain	Simulation study (Markov)		CUA, CBA	Society	Smokers, 16 years or older *n* = 3.1 Mio (Cohort) Total estimated adult smokers, motivated to quit within the next 12 months	I1: Usual care: minimal physician counseling + self-help materials I2: Proactive telephone counseling I3: NRT; I4: Var; I5: Bupr vs. unaided smoking cessation	12 months (cycle)
Wu, 2014 UK	RCT, Simulation study (Markov)		CUA	Healthcare	Smokers between 18–65 years *n* = 6,911, follow-up: 77% Invitation to a random sample of GP patients	Computer-based intervention (ESCAPE) vs. general information	6 months
Reimbursement							
Cadier, 2016 France		Simulation study (Markov)	CEA	Healthcare	Smokers between 15–75 years *n* = 1,000 (Cohort)	Reimbursement of medication vs. usual care (€50 reimbursement)	12 months (cycle)
Chevreul, 2014 France		Simulation study (Markov)	CEA	Healthcare	Smokers between 15–75 years	Reimbursement of medication and 6 individual counseling sessions vs. usual care (€50 reimbursement)	12 months (cycle)
Gebreslassie, 2023 Sweden		Simulation study (Markov)	CUA	Healthcare and society	Smokers, 16 years or older	Reimbursement of NRT for 3 months vs. usual care	5 years (cycle)
Kaper, 2006 Netherlands		RCT	CEA, CUA	Society	Smokers, 18 years or older, average age: 40 *n* = 1,266 (I: 634, C: 632), follow-up: I: 81.5%, C: 73.1% Random sample	Reimbursement (NRT, Burp, counseling) for 6 months vs. usual care: no reimbursement	12 months
Vemer, 2010 Netherlands		Simulation study (Microsimulation)	CEA, CUA	Healthcare	Smokers, 16 years or older *n* = 3.8 Mio. (cohort) Estimated smokers in the Netherlands	Reimbursement (NRT, Burp, counseling) for 6 months vs. usual care: no reimbursement	12 months (cycle)
Awareness building							
Gilbert, 2017 Wu, 2018 UK		RCT, simulation study (Markov)	CUA	Healthcare	Smokers 16 years or older, willing to quit, have not attended NHS Stop Smoking Services (SSS) within the last 12 months, average age: 49.3 *n* = 4,383 (I: 2,635, C: 1,748), follow-up: 76.9% Invitations to participate sent to GP patients	NHS SSS Start to quit (personalized letter by GP + invitation to join a “come and try” meeting) ->proactive recruitment vs. standard letter by GP to inform about local SSS	6 months
Kotz, 2011 UK		Observational study, simulation study (Microsimulation)	CEA	Healthcare	Smoking Toolkit Study (STS) participants; daily or occasional smokers *n* = 3,981 (I: 1,309, C: 2,672) Cluster sampling	No Smoking Day: annual nationwide marketing campaign vs. usual care	12 months (cycle)
Nèmeth, 2018 Hungary		Simulation study (Markov)	CEA, CUA, CBA	Society	Smokers, 18 years or older	I1: Nationwide marketing campaign I2: Double group therapy and proactive telephone counseling I3: Combination I1 + I2 vs. usual care	12 months (cycle)

*C *control group,* CBA *Cost-Benefit Analysis,* CEA *Cost-Effectiveness Analysis,* CESAR *Cost-effectiveness of a Tobacco Cessation Care Service in the Community Pharmacy,* CUA *Cost-Utility Analysis,* GP *General practitioner,* I *Intervention Group,* SSS *NHS Stop Smoking Services,* SCS *Smoking Cessation support,* STS *Smoking Toolkit Study,* UK *United Kingdom

## Health economic outcomes

### Cost-effectiveness of face-to-face counseling

Of the 21 reported ICERs, 18 show cost-effectiveness below the EUR 25,000/QALY threshold. Considering a willingness to pay of EUR 50,000/QALY, all studies can be classified as cost-effective. Olsen et al. further show that all forms of nicotine cessation are below the EUR 25,000/LYS threshold. The ICER for men is EUR 1,718/LYS, compared to 2,211 EUR/LYS for women. In an overall analysis of the included studies, the ICERs range between EUR − 223,449/QALY [49] and EUR 14,011.65/QALY [40]. Trapero-Bertran et al. do not report ICER values directly, but their sensitivity analysis indicates that the Spanish Smoking Cessation Program dominates the control group, indicating it is cost-saving and more effective [47].

### Cost-effectiveness of digital and telephone counseling

The costs of digital counseling interventions are moderate compared to face-to-face counseling interventions. Those studies that use QALYs as an outcome emphasize that all interventions, except text messages, show QALY gains between 0.001 and 0.02 from an individual perspective. Text-based interventions themselves show inferiority compared to standard care in terms of outcomes. The evidence synthesis shows that the ICERs for web, text and computer-based counseling interventions vary between EUR − 332,320/QALY (text-based intervention, strongly inferior) and EUR 79,130/QALY (video-based intervention, weakly inferior) [45]. Overall, two ICERs are above the threshold value of EUR 25,000/QALY and are therefore not cost-effective. A further two ICERs show a negative sign, which indicates that the outcomes are inferior to standard care.

The ICERs for telephone counseling vary between EUR 191.90/LYS [42] and EUR 462.31/LYS [46]. The study by Feenstra et al. reports a higher incremental cost-effectiveness of telephone counseling (ICER EUR 2,721.51 per quitter) compared to counseling in combination with nicotine replacement therapy (NRT) (ICER: EUR 2,885.64-4.64,927.23 per quitter), but a lower incremental cost-effectiveness ratio compared to minimal face-to-face counseling (EUR 736.25 per quitter) [32].

### Cost-effectiveness of reimbursement

Five studies on the reimbursement of smoking cessation therapies were considered in this review. The included studies list the total costs of reimbursement. The analysis of the 12 reported QALY gains from reimbursement shows that on average 0.04 QALYs (SD = 0.05) are gained per person. In the included studies, the ICER varies between EUR − 34,506/QALY and EUR 31,481/QALY [34]. The incremental net benefits (INB) of the studies suggest that around 13% of the ICERs are above the threshold of EUR 25,000/QALY and are therefore not cost-effective. Considering the threshold value of EUR 50,000/QALY, all interventions are cost-effective.

### Cost-effectiveness of awareness Building

Overall, the studies reports that awareness-building is dominant over no intervention [39]. Thus, all awareness-building interventions are located in the northeastern quadrant of the cost-effectiveness quadrant when they are calculated for a short-term period of six months. If the CEA uses a lifetime perspective, the ICER is located in the southeastern quadrant of the CE-plane. Given the very low ICERs in the UK studies, it can be concluded that these approaches are low-cost and effective. A summary of all health economic parameters is presented in Table 3.

**Table 3:** Economic parameters

Author, year Study design	Interventions costs in EUR	Discount rate	QALY/LYS	QoL	% Quitters	Difference I vs. C	ICER (converted 2023), ROI reported	ICER calculated from study data and converted (2023)	Net benefit calculated and converted (2023) in EUR
**Face-to-face counseling**									
Bauld, 2011 Observational study, simulation study	*Individual counseling*: *I: 139*,*47 p.p* *C (self quit): 0* *Group therapy*: *I: 649.69 p.p.* *C (self quit): 0*	*3.5% (Effectiveness)*	*Individual counseling*: *I: 15.2 QALY* *C: 15.17 QALY* *Group therapy*: *I: 15.25 QALY* *C: 15.17 QALY*	*EQ-5D*	*12-months CA* *Group therapy: 6.3%* *Individual counseling: 2.8%*	*Individual counseling: 0.03 QALY* *Group therapy: 0.08 QALY*	*ICER EUR/Quitter* *Individual counseling: 7*,*800* *Group therapy: 16*,*242.28* *ICER EUR/QALY* *Individual counseling: 4*,*590* *Group therapy: 8*,*474.23*	*ICER EUR/QALY* *(Lifetime)* *Individual counseling: 4*,*649* *Group therapy: 8*,*121.13*	*Individual counseling: 139*,*47* *Group therapy: 649.69*
Feldman, 2019 RCT, simulation study	*HIT: 140*,*910.68* LIT: 33,631.13 (*n* = 300)	*3% (cost and effectiveness*	*n.a.*	*EQ-5D*	*CA 6 months* *HIT: 27 (18%)* *LIT: 14 (9%)* *SA 5–8 Years* *HIT: 17 (16%)* *LIT: 7 (3%)*	*Short-term (CA 6 months): 7.44 QALY* *Long-term (SA 5–8 years): 5.71 QALY*	*ICER EUR/QALY* *Short-term: 1*,*105.50* *Long-term: 4*,*559.30*	*ICER EUR/QALY* *Short-term: 1*,*105.84* *Long-term: 4*,*559.49*	*Short-term: 8*,*227.45 * *Long-term: 26*,*034.71*
Gòmez-Martinez, 2024 Non-randomized controlled study	*I (behavioral therapy): 1.100*,*93 p.p. (society)*,* 657.94 (healthcare)* C (usual care): 1,591.78 p.p. (society), 972.35 p.p. (healthcare)	*n.a.*	*I: 0.88 QALY* C: 0.85 QALY	*EQ-5D*	*CA 12 months* *I: 432 (54.3%)* *C: 103 (37.1%)*	*0.03 QALY* *329 Quitters*	*ICER EUR/Quitter* *Society + healthcare*: *dominant* *ICER EUR/QALY* *Society + healthcare*: *dominant*	*ICER EUR/Quitter* *Society: −1.49* *healthcare: −0.96* *ICER EUR/QALY (12 months)* *Society: −16*,*361.67* *healthcare: −10*,*480.33*	*Society: −490.85* *healthcare: −314.41*
Nohlert, 2013 RCT, simulation study	*HIT: 133*,*777.12 (n = 300)* LIT: 31,928.24 (*n* = 300)	3% (cost and effectiveness	HIT: 13.85 QALY LIT: 9.91 QALY	n.a.	CA 6 months HIT: 27 (18%) LIT: 13 (9%) 7-day PPA HIT: 35 (23%) LIT: 24 (16%)	*7-day PPA: 3.94 QALY* *CA 6 months: 3.94 QALY*	*ICER EUR/Quitter* *HIT: 3*,*169.63* *LIT: 1*,*105.12* *ICER EUR/QALY* *Society* *7-day PPA: 11*,*646.27* *CA 6 months: 6*,*448.56*	*ICER EUR/QALY* *Society* *7-day PPA: 14*,*011.65* *CA 6 months: 7*,*759.05*	*7-day PPA: 55*,*205.89 * *CA 6 months: 40*,*812.62*
Olsen, 2006 Simulation study	*n.a.*	*3.5% (cost and effectiveness*	*n.a.*	*n.a.*	*SCT: 31% * *No intervention: 7% (estimated)*	*LYS* *Reference case: 0.35* *Males: 0.4 * *Females: 0.34 * *Light smokers: 0.41* *Heavy smokers: 0.34 * *Pharmacy: 0.35 * *Hospital: 0.41 * *25–34 years: 0.05 * *35–54 years: 0.24 * *55 years and older: 0.67*	*ICER EUR/LYS* *Reference case: 2*,*206.65* *Males: 1*,*771.17* *Females: 2*,*211.53* *Light smokers: 1*,*810.17* *Heavy smokers: 2*,*213.15* *Pharmacy: 2*,*211.53* *Hospital: 1*,*719.17* *25–34 years: 15*,*682.18* *35–54 years: 3*,*223.86* *55 years and older: 1*,*093.58*	*ICER EUR/LYS * *Reference case: 2*,*089.20* *Males: 1*,*718.35* *Females: 2*,*150.64* *Light smokers: 1*,*751.75* *Heavy smokers: 2*,*093.29* *Pharmacy: 2*,*154.2* *Hospital: 1*,*688.34* *25–34 years: 13*,*486.8* *35–54 years: 3*,*019.67* *55 years and older: 1*,*074.39*	*Reference case: 731.22 * *Males: 687.34 * *Females: 731.22 * *Light smokers: 718.22 * *Heavy smokers: 711.72 * *Pharmacy: 753.97 * *Hospital: 692.22 * *25–34 years: 674.34 * *35–54 years: 724.72 * *55 years and older: 719.84*
Salize, 2009 Cluster RCT	*I1: 22.45 p.p. GP training + renumeration (TI)*, *I2: 62 p.p. GP training + medication (TM)*, *I3: 79.92 p.p. (TI/TM)*	*n.a.*	*n.a.*	*n.a.*	*PPA 12 Months* *TI: 5 (3.5%)* *TM: 12 (12.1%)* *TI/TM: 32 (14.6%)* *Usual care: 2 (2.7%)*	*n.a.*	*ICER EUR/Quitter* *TM: 6.57* *TI/TM: 6.67*	*ICER EUR/Quitter* *TM: 7* *TI/TM: 7*	*n.a.*
Virtanen, 2017 Cluster RCT, simulation study	*I (counseling): 620.53 p.p.* *C (usual care): 0* *Cost savings*: *I: 766*,*102.75* *C: 614*,*157.27*	*3% (cost and effectiveness*	*I: 5.42 QALY* *C: 4.74 QALY*	*EQ-5D*	*PPA* *I: 8%* *C: 8%*	*0.68 QALY*	*ICER EUR/QALY* *population-based + Markov: dominant*	*ICER EUR/QALY: −223*,*449.23*	*ICER EUR/QALY (training costs): 10.15* *ICER EUR/QALY (total cost): 912.54* *ICER EUR/QALY (incl. Cost-savings): * *−151*,*945.48*
***Digital Counseling***									
Feenstra, 2005 Simulation study	*Telephone counseling (TC): 116*,*175.21* *Minimal counseling (MC): 35*,*003.31* *MC + NRT: 270*,*236.16* *Intensive counseling (IC) + NRT: 270*,*236.16* *IC + Bupr: 553*,*509.51* *C (Usual care: 1*,*871.92* *(n = 1,000)*	4% (cost and effectiveness	*n.a.*	*n.a.*	*12 months PA* *(estimated)* *TC: 7.6% * *MC: 7.9%* *MC + NRT: 12.7%* *IC + NRT: 15.1%* *IC + Bupr: 17.2% * *C: 3.4%*	*Quitters* *TC: 42* *MC: 45* *MC + NRT: 93 * *IC + NRT: 117* *IC + Bupr: 138*	*EUR/Quitter* *TC: 2*,*716.77* *MC: 745.46* *MC + NRT: 2*,*899.00* *IC + NRT: 4*,*920.01* *IC + Bupr: 3*,*992.33* *ICER EUR/LYS* *TC: 2*,*319.20* *MC + NRT: 2*,*981.82* *IC + NRT: 10*,*270.73* *IC + Bupr: 7*,*123.25* *ICER EUR/QALY* *TC: 2*,*716.77* *MC + NRT: 2*,*319.20* *IC + NRT: 8*,*117.19* *IC + Bupr: 5*,*632.34*	*ICER EUR/Quitter* *(Lifetime)* *TC: 2*,*721.51* *MC: 736.25* *MC + NRT: 2*,*885.64* *IC + NRT: 4*,*927.23* *IC + Bupr: 3*,*997.37*	*TC: 114*,*303.29* *MC: 33*,*131.39* *MC + NRT: 26*,*8364.24* *IC + NRT: 576*,*486.13* *IC + Bupr: 551*,*637.59*
Rasmussen, 2013 Observational study, simulation study	*I (TC): 259*,*652.74*	3% (effectiveness	*I: 866 LYS*	*n.a.*	*CA12: 19%*	*213 LYS*	*ICER EUR/LYS* *PPA12: 192.47* *CA12: 299.24*	*ICER EUR/LYS* *PPA12: 191.90* *CA12: 299.83*	*259*,*652.74*
Smit, 2013 RCT	*Web + Coun: 2*,*139.02 p.p.* *Web: 1*,*389.54 p.p.* *C (usual care): 1*,*040.79*	*n.a.*	*Web + Coun: 0.86 QALY* *Web: 0.83 QALY* *C: 0.84 QALY*	*EQ-5D*	*PA* *Web + Coun: 14 (18.6%)* *Web: 20 (15.2%)* *C: 12 (10.1%)*	*Web + Coun: 0.02 QALY* *Web: −0.01 QALY*	*ICER EUR/QuitterWeb + Coun: dominant* *(- EUR54*,*911.48)* *Web: 6*,*975.06* *ICER EUR/QALY* *Web + Coun: 54*,*911.48* *Web: dominant (- EUR34*,*875.28)*	*ICER EUR/QALY* *Web + Coun: 54*,*911* *Web: * *−34*,*875*	*Web + Coun: 1*,*098.23* *Web: 348.75*
Stanczyk, 2014 RCT	*Video-based: 6*,*513.18 p.p.* *Text-based: 7*,*098.69 p.p.* *C (Generic info): 6*,*434.05 p.p.*	*n.a.*	*Video-based: 0.83 QALY* *Text-based: 0.83 QALY* *C: 0.83 QALY*	*EQ-5D*	*Video-based: PA: 66 (9.9%)*,* PPA: 119 (17.8%)* *Text-based: PA*: *52 (7.3%)*,* PPA: 125 (17.7%)* *C: PA: 46 (6.4%)*,* PPA: 116 (16.2%)*	*Video-based: 0.001 QALY* *Text-based: −0.002 QALY*	*ICER EUR/QALY* *Video-based: 79*,*123.44* *Text-based: dominant*	*ICER EUR/QALY* *Video-based: 79*,*130* *Text-based: −332*,*320*	*Video-based: 79.13* *Text-based: 664.64*
Tomson, 2004 Observational study	*n.a.*	*3% (costs)*,* 5% (effectiveness)*	*n.a.*	*n.a.*	*TC* *PPA12: 274 (24%)*	*n.a.*	*EUR/Quitter: 1*,*052 − 1*,*360* *EUR/LYS: 311–401*	*ICER EUR/LYS * *TC: 462.31*	*n.a.*
Trapero-Bertran, 2018 Simulation study	*n.a.*	3% (cost and effectiveness	*n.a.*	*EQ-5D*	*Quitters per 1*,*000 smokers* *(estimated)* *Usual care: 18.18* *TC: 18.28* *NRT: 19.68*	*n.a.*	*ICER EUR/QALY* *TC 10 years: 10*,*052.51*,* Lifetime: dominant* *NRT: 10 years 30*,*046.50*,* Lifetime: dominant* *ROI (per 1 EUR)* *TC 10 years: 0.70*,* Lifetime: 1.87* *NRT 10 years: 0.43*,* Lifetime: 1.17*	*n.a.*	*n.a.*
Wu, 2014 RCT, Simulation study	*6 months*: *I (tailored text): 109.53 p.p.* *C (generic text): 95.23 p.p.*	3.5% (cost and effectiveness	*I: 0.397 QALY* *C: 0.396 QALY*	*EQ-5D*	*3-months PA* *I: 5.1%* *C: 3.5%*	*6-months: 0.0006 QALY* *Lifetime: 3 QALY*	*ICER EUR/QALY * *6-months: 24*,*196.99* *Lifetime: 16*,*263.22*	*ICER EUR/QALY * *6-months: 23*,*833.33* *Lifetime: 18*,*862.5*	*6-months: 14.30* *Lifetime: 56*,*587*
***Reimbursement***									
Cadier, 2016 Simulation study	*n.a.*	*3% (cost and effectiveness)*	*n.a.*	*n.a.*	*Reimbursement: 7.04% (estimated)* *Usual care: 2.6% (estimated)*	*n.a.*	*ICER EUR/LYS * *15–24 years* *Males: 9*,*832.86/Females: 11*,*788.48* *25–34 years* *Males: 5*,*334.39/Females: 6*,*521.52* *35–44 years* *Males: 3*,*540.34/Females: 4*,*408.56* *45–54 years* *Males: 3*,*654.13/Females: 4*,*293.36* *55–64 years* *Males: 5*,*689.83/Females: 6*,*104.27* *65–74 years* *Males: 11*,*059.34/Females: 10*,*608.36*	*ICER EUR/LYS (Lifetime)* *15–24 years* *Males: 9*,*832.86/Females: 11*,*788.48* *25–34 years* *Males: 5*,*334.39/Females: 6*,*521.52* *35–44 years* *Males: 3*,*540.34/Females: 4*,*408.56* *45–54 years* *Males: 3*,*654.13/Females: 4*,*293.36* *55–64 years* *Males: 5*,*689. 83/Females: 6*,*104.27* *65–74 years* *Males: 11*,*059.34/Females: 10*,*608. 26*	*n.a.*
Chevreul, 2014 Simulation study	*n.a.*	*3% (cost and effectiveness*	*n.a.*	*n.a.*	*Reimbursement: 7.04% (estimated)* *Usual care: 2.6% (estimated)*	*n.a.*	*ICER EUR/LYS: 2*,*684.75*		*n.a.*
Gebreslassie, 2023 Simulation study	*Society*: *Total: 53*,*032.51* *16–30 years: 80*,*879.46* *31–44 years: 86*,*891.46* *45–64 years* *62*,*201.5* *65 years and older: 17*,*131.88* *Healthcare*: *Total: 10*,*106.46* *16–30 years: 9*,*277.38* *31–44 years* *10*,*466.81* *45–64 years* *11*,*225.49* *65 years and older: 8*,*902.21*	*3% (cost and effectiveness*	*Society*: *Total: 13.58 QALY* *16–30 years: 24.33 QALY* *31–44 years: 20.38 QALY* *45–64 years: 13.94 QALY* *65 years and older: 6.15 QALY* *Healthcare*: *Total: 13.61 QALY* *16–30 years: 24.33 QALY* *31–44 years: 20.48 QALY* *45–64 years: 13.97 QALY* *65 years and older: 6.15 QALY*	*EQ-5D*	*Reimbursement vs. NRT: 11.9% (estimated)* *Reimbursement: vs. usual care: 7.7% (estimated)*	*Society: QALY* *All age groups: 0.03 * *16–30 Years: 0.02 * *31–44 Years: 0.02 * *45–64 Years: 0.04 * *65 years and older: 0.04 * *Healthcare*: *All age groups: 0.04 * *16–30 Years: 0.01 * *31–44 Years: 0.02* *45–64 Years: 0.04 * *65 years and older: 0.05*	*ICER EUR/QALY* *Society:* *All age groups: dominant* *16–30 years: dominant* *31–44 years: dominant* *45–64 years: dominant* *65 years and older: 6*,*049.89* *Healthcare:* *All age groups: 13*,*413.52* *16–30 years: 30*,*960.41* *31–44 years: 20*,*913.24* *45–64 years: 11*,*943.41* *65 years and older: 10*,*961.48*	*ICER EUR/QALY* *(Lifetime)* *Society:* *All age groups: −8*,*090* *16–30 years: −26*,*308* *31–44 years: −34*,*506.5* *45–64 years: 10*,*583.5* *65 years and older: 6*,*785.5* *Healthcare:* *All age groups: 11*,*996.25* *16–30 years: 49*,*653* *31–44 years: 24*,*131.5* *45–64 years: 31*,*481.75* *65 years and older: 9*,*745.2*	*Society*: *All age groups: −242.70* *16–30 years: −526.16* *31–44 years: −690.13* *45–64 years: −423.34 * *65 years and older: 271.42 * *Healthcare: * *All age groups: 479.85 * *16–30 years: 496.53 * *31–44 years: 482.63 * *45–64 years: 1*,*259.27 * *65 years and older: 487.26*
Kaper, 2006 RCT	*I (reimbursement): 326,547.69 (n = 634)* *C (usual care): 296,846.50 (n = 634)*	*4% (effectiveness)*	*I: 22.4 QALY* *C: 10.6 QALY*	*n.a.*	*Quitters after 6-months* *I: 49 (7.8%)* *C: 35 (5.5%)* *Quitters after 12 months* *I: 35 (5.5%)* *C: 18 (2.8%)*	*11.8 QALY*	*ICER 6 Months* *EUR/Quitter: 1*,*796.08* *ICER Lifetime* *EUR/QALY: 2*,*894.93*	*ICER EUR/QALY* *(Lifetime): 2.894*,*13*	*29*,*701.19*
Vemer, 2010 Simulation study	*n.a.*	*4% (costs)*,* 1.5% (effectiveness)*	*n.a.*	*n.a.*	*PA 12 months (RCT Kaper 2006)* *Reimbursement: 35 (5.5%)* *Usual care: 18 (2.8%)*	*20 years: 11*,*200 QALY* *Lifetime: 54*,*600 QALY* *20 years: 9*,*200 LYS* *Lifetime: 67*,*700 LYS*	*ICER EUR/LYS* *20 years: 7*,*281.21* *Lifetime: 5*,*457.11* *ICER EUR/QALY* *20 years: 5*,*973.94* *Lifetime: 6*,*764.38*	*ICEREUR/QALY*: *20 years: 5*,*958* *Lifetime: 6*,*762.43* *ICER EUR/LYS*: *20 years: 7*,*253.45 Lifetime: 5*,*453.89*	*20 years: 66*,*731*,*744.78 * *Lifetime: 369*,*228*,*720*
***Awareness-building interventions***									
Gilbert, 2017 Wu, 2018 RCT, simulation study	*I (personalized letter): 1*,*212.20 p.p.* *C (generic letter): 1*,*067.05 p.p.*	3.5% (cost and effectiveness)	*I: 0.382* *C: 0.380*	*EQ-5D*	*PPA6* *I: 236 (9.0%)* *C: 97 (5.6%)* *PA 3-months* *I: 150 (5.7%)* *C: 60 (3.4%)*	*QALY* *6 Months: 0.002* *Lifetime: 0.196*	*ICER EUR/QALY* *6 months: * *94*,*744.50* *Lifetime: dominant* *(−599.72)*	*ICER EUR/QALY* *6 months*: *156*,*310* *80*,*638.89* *Lifetime: −657.55*	*6 months: 145.15* *Lifetime: −116.43*
Kotz, 2011 Observational study, simulation study	*n.a.*	*3.5% (effectiveness)*	*n.a.*	*n.a.*	*Marketing campaign: 0.07% (estimated)*	*0.001 QALY*	*ICER EUR/LYS* * < 35 years: 195.28* *35–44 years: 140.52* *45–54 years: 130.18* *55–64 years: 166.51*	*n.a.*	*0.83 EUR p.p.*
Nèmeth, 2018 Simulation study	*n.a.*	*3.7% (cost and effectiveness)*	*n.a.*	*EQ-5D*	*Quitters per 1*,*000 smokers (estimated)* *Marketing campaign (MRC): 0.6295* *Double behavioral counseling (DBC): 10.4108* *MC + DBC): 10.7098* *Usual care: 10.3280*	*n.a.*	*ROI (savings healthcare)* *MRC: 1.9084* *DBC: 3.1045* *MRC + DBC: 2.0767* *ROI (savings healthcare and health benefits)* *MC: 20.8036* *DBC: 33.8423* *MRC-DBC: 22.6387* *ICER EUR/LYS* *MRC: dominant* *DBC: dominant* *MRC-DBC: dominant* *ICER EUR/QALY* *MRC: dominant* *DBC: dominant* *MRC + DBC: dominant*	*n.a.*	*n.a.*

*C* Control group, *CA* continued abstinence, *Coun* Couunseling, *DBC* Double behavioral counseling, *EQ-5D* European Quality Index in fife dimensions, *HIT* High-intensity training, *I* Intervention group, *IC* Intensive counseling, *ICER* Incremental Cost-Effectiveness Ratio, *LIT* Light-intensity training, *LYS* Life Years Saved, *MC* Minimal counseling, *MRC* Marketing campaign, *n.a.* not applicable, *NRT* Nicotine Replacement Therapy, *PA* Prolonged Abstinence, *PPA* Point Prevalence Abstinence, *QALY* Quality-adjusted Life Years, *QoL* Quality of Life, *RCT* Randomized Controlled Trial, *ROI* Return on Investment, *SA* Sustained Abstinence, *SCT* Smoking Cessation Treatment, *TC* Telephone counseling, *TI* training + renumeration, *TIM* training + medication

### Narrative Evidence-synthesis

**Fig. 2:**
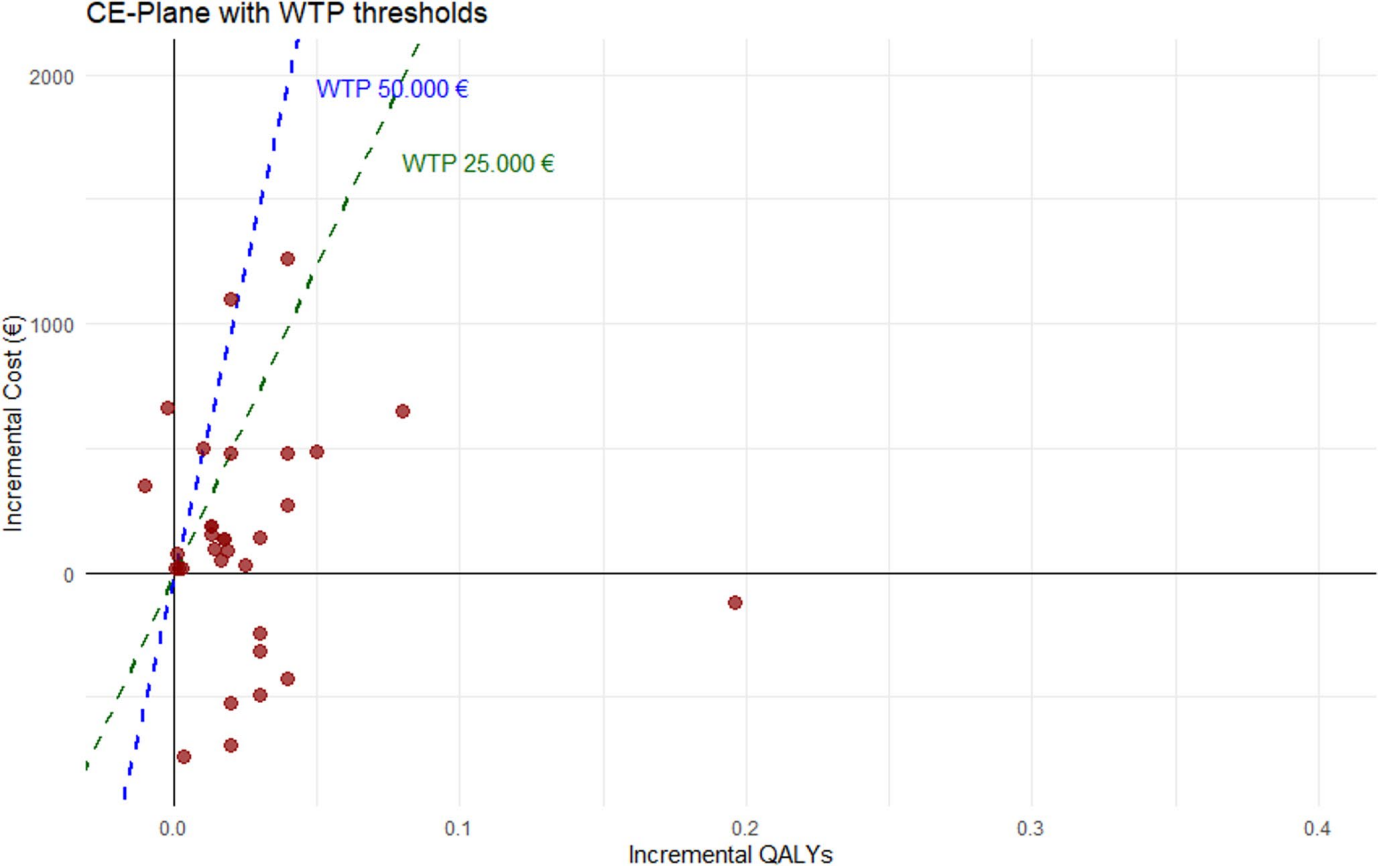
CE-Plane EUR/QALY

Overall, the cost-effectiveness analyses reveal a clear trend. 30 of 39 ICERs (76.9%) demonstrate cost-effectiveness, with 21 ICERs positioned in the northeastern quadrant, indicating weak dominance below the EUR 25,000/QALY threshold. Meanwhile, nine ICERs show strong dominance in the southeast quadrant [34–36, 49, 51]. Among the inferior ICERs, two are strongly dominated by non-intervention [44, 45], while seven are dominated due to exceeding the EUR 25,000/QALY threshold [34, 35, 44, 45, 51]. A graphical representation of the ICERs in EUR/QALY can be found in Fig. 2.

Overall, two studies indicate that the control group dominates the intervention [44, 45]. Most studies that report negative ICERs conclude that nicotine cessation is cost-saving and generates QALYs [34–36, 49]. Thus, nine of the 28 ICERs (or 28%) exhibit a strong dominance of tobacco cessation. However, it is important to note that four of these nine ICERs come from a single study [34], which reports high variability in ICER estimates and cost-effectiveness across age groups. This study highlights that the 16–30 year-old and 45–64 year-old exhibit high positive ICERs, exceeding the EUR 25,000 per QALY threshold. However, the same study suggests that, from a societal perspective, cost savings can still be generated across all age groups under 65 years. The results suggest that interventions offering personal counseling generally dominate the control group [29, 33, 36, 40, 49], with two of the 14 ICERs even demonstrating strong dominance [36]. Digital counseling, however, presents mixed results. Two studies report ICERs exceeding EUR 25,000/QALY [45]. One study reports strong inferiority of a text-based digital tool [44]. Another study suggests that digital solutions maybe cost-effective under the EUR 25,000/QALY threshold [50]. Across all studies, the ICERs ranging from EUR − 332,320/QALY to EUR 156,310/QALY. When categorizing results by intervention types, the ICERs vary broadly (Table 4). In terms of reimbursement, there is a trend toward cost-effectiveness in the north-east quadrant, while three measures are located in the south-east quadrant, and five exceed the EUR 25,000/QALY threshold [34, 35].

**Table 4:** Health economic outcomes over the intervention groups

	Median Cost difference per Capita (I-C) (SD)	Median Effectiveness gained per Capita (I-C) (SD)	ICER Min.	ICER Max.
Face-to-face Counseling	*EUR 136.04 (EUR 359.52)*	*0.03 QALY (0.23)*	*EUR −223.449/QALY*	*EUR 14*,*012/QALY*
	*EUR 719.03 (EUR 23.85)†*	*0.34 LYS (0.15)†*	*EUR 1*,*074/LYS†*	*EUR 13*,*487/LYS†*
Digital/telephone counseling	*EUR 213.94 (EUR 398.89)*	*0.01 QALY (0.01)*	*EUR − 332*,*320/QALY*	*EUR 79*,*130/QALY4*
	*n.a.*	*n.a.*	*EUR 192/LYS*	*EUR 13*,*487 LYS*
Reimbursement	*EUR 97.17 (EUR 509.34)*	*0.01 QALY (0.02)*	*EUR − 34*,*506/QALY*	*EUR 49*,*653/QALY*
	*n.a.*	*n.a.*	*EUR 1*,*106/LYS*	*EUR 14*,*012/LYS*
Awareness building	*EUR − 122.65 (EUR 8*,*80) (Lifetime)* ^*††*^	*0.20 QALY (0)* ^*††*^	*EUR − 658/QALY*	*EUR − 594/QALY*
	*EUR 150.73 (EUR 5.58) (6 months)* ^*††*^	*0.00 QALY (0.00)* ^*††*^	*EUR 72*,*575/QALY*	*EUR 156*,*310/QALY*

*C *Control group,* I *Intervention group,* n.a. = *not applicable, ^*†*^ referred to one single study [40], ^*††*^ referred to one single study [34, 50]

**Fig. 3:**
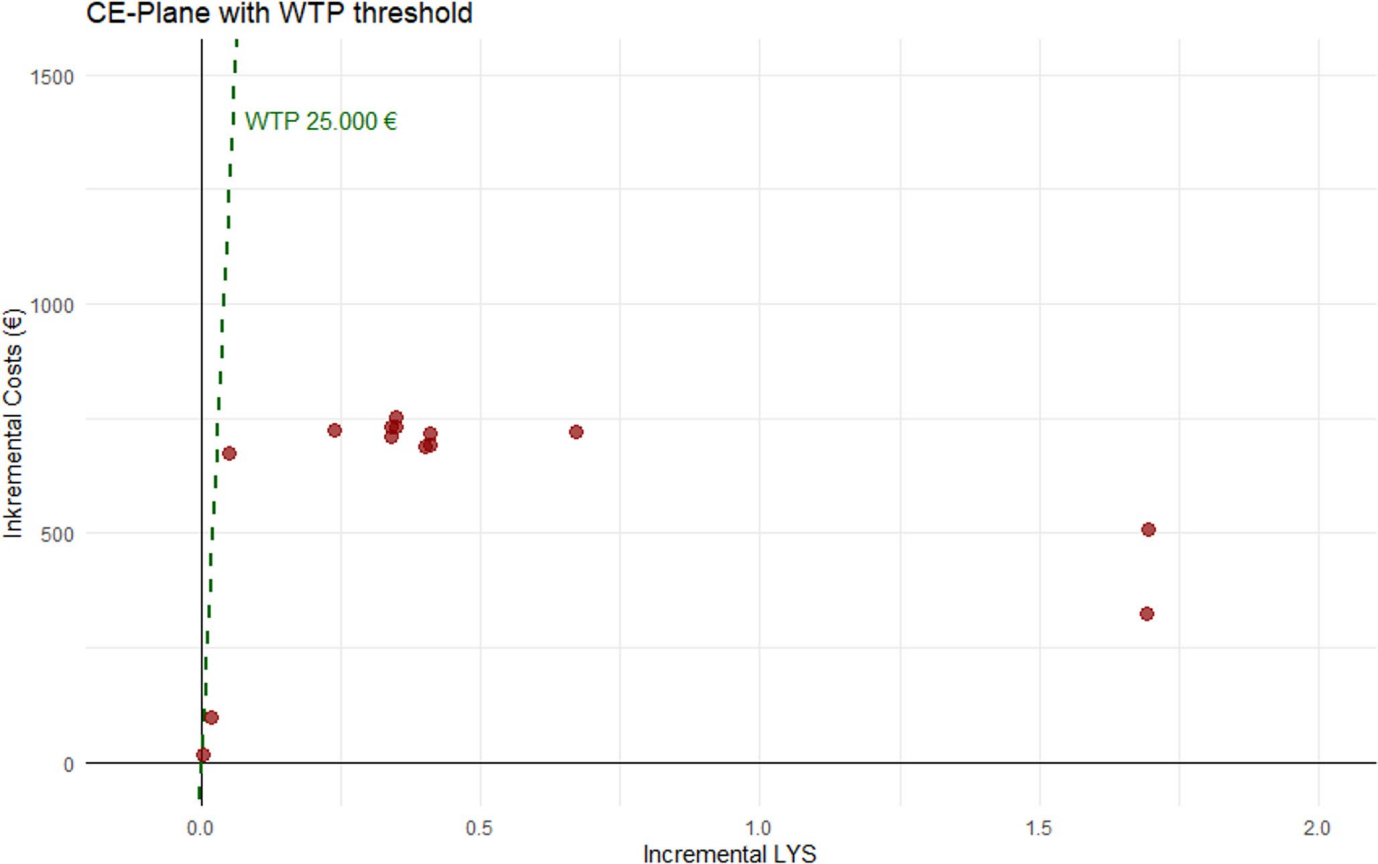
CE-Plane EUR/LYS

Figure 3 presents the ICERs calculated for the life years saved (LYS). The ICERs ranging from EUR 192/LYS [42] to EUR 17,908/LYS [46]. Here, all ICERs are positioned in the northeastern quadrant of the CE-plane, indicating that 100% of studies measuring LYS report dominance over standard care. Notably, most reimbursement studies use LYS as a measurement metric.

### Influence of perspective on cost-effectiveness estimates

Most studies report cost-effectiveness from both a societal and a healthcare perspective, suggesting that the choice of perspective does significantly impact the overall cost-effectiveness conclusions. For studies using the healthcare perspective, ICER results range from EUR − 34,875/QALY to EUR 156,310/QALY. When applying the societal perspective, studies report lower ICERs, which can be attributed to the inclusion of productivity losses due to smoking and the recognition of avoided societal costs as cost-savings [33, 34, 36, 40, 45, 48, 49]. From the societal perspective, the calculated ICERs range from EUR − 332,320/QALY to EUR 79,130/QALY and have significant lower ICER than the CEA applying the healthcare perspective. This variability reflects the fact that many studies, using the societal perspective, consider cost-savings resulting from successful smoking cessation. The potential savings from avoided social costs of smoking are estimated at between EUR 484 and EUR 2,003 per person per year [40]. Reisinger et al. report similar results in a systematic review [52]. Feldman et al. and Nohlert et al. showed that, from a lifetime perspective, the highest cost savings generated by avoided smoking-related productivity loss occur in males aged 40 to 46 years of age (approx. 13.600 EUR per person) and are lowest in females over 65 years of age [33, 40]. Virtanen et al. and Vemer et al. calculated total cost savings in a simulation model ranging from EUR 199,000 to EUR 297.000 [48, 49].

### Sensitivity analyses

All included studies conduct sensitivity analyses to assess the uncertainty of the ICER estimate [29–51]. Eleven studies perform one-way sensitivity analyses and vary several parameters of the models [29, 32, 33, 37, 38, 40–42, 46, 48, 49]. These studies focus on key estimation factors, such as relapse rates, additional users, cost variations, and discount rates, as well as various outcome parameters and their discount rates. Five studies estimate uncertainty using non-parametric bootstrapping methods, which include only complete cases [35, 36, 44, 51]. Eight studies perform probabilistic sensitivity analyses, either independently or in combination with univariate or multivariate sensitivity analyses [30, 31, 33, 34, 39, 40, 47, 50]. These probabilistic sensitivity analyses largely follow established recommendations such as those of Briggs et al. [53], ensuring appropriate distribution models. Two studies used the same model for their sensitivity analyses [33, 54]. The sensitivity analyses suggest that the considered uncertainties have minimal impact on the health economic evaluation of the interventions. A summary of all sensitivity analyses is shown in the Table 5.

**Table 5:** Summary of sensitivity analyses

Author	Sensitivity analysis form	Variation	Result
** Face-to-face counseling**			
Bauld	One-Way sensitivity analysis	1. QALY not discounted 2. Self-reported quit rates incl. 3. Small cost to the NHS (due to self-quitting) 1. Longer costs in smoking-related disease	Approx. 100% probability of cost-effectiveness under EUR 25,000/QALY
Feldman	One-way and multivariate analysis and Probabilistic sensitivity analysis	Univariate analysis: 1. Disease risk: +100%, −50% 2. Death risk (+/- 10%) 3. Risk fractions of disease after quitting: +/- 0.1 4. All disease costs: +25% 5. QoL weight 1 during healthy years. 2. PSA: Average of differences in cost and QALY between smokers and quitters	Approx. 100% probability of cost-effectiveness under EUR 25,000/QALY for HIT; approx. 0% probability of cost-effectiveness under EUR 25,00/QALY for LIT
Gómez Martínez	Deterministic sensitivity analysis: 1,000 repetitions of CEA with just completed cases	n.a.	88.1% of ICER replication in south-east quadrant of the CE-plane, approx. 96% probability of cost-effectiveness under EUR 25,000/QALY
Nohlert	One-way and multivariate sensitivity analysis and Probabilistic sensitivity analysis	Univariate analysis: 1. Disease risk: +100%, −50% 2. Death risk (+/- 10%) 3. Risk fractions of disease after quitting: +/- 0.1 4. All disease costs: +25% 5. QoL weight 1 during healthy years. PSA: Average of differences in cost and QALY between smokers and quitters	Approx. 80% probability of cost-effectiveness under EUR 25,000/QALY (High Intensity training); 20% probability of cost-effectiveness under EUR 25,000/QALY (Low Intensity training)
Olsen	One-Way and Probabilistic sensitivity analysis	Cessation start = Lognormal distribution Abstinence rate = Beta distribution Costs = Log-normal distribution	Approx. 100% probability of cost-effectiveness under EUR 25,000/LYS (age > 34 years); approx. 80% probability of cost-effectiveness under EUR 25,000/LYS (age < 35 years)
Salize	Multivariate sensitivity analysis	1. Variation of point prevalence of quitters 2. GP costs: 0 EUR 3. Tutor costs reduction: −700 EUR	Approx. 95% cost-effectiveness under 9.80 EUR per additional quitter (compared to treatment-as-usual)
Virtanen	One-Way sensitivity analysis	1. Proportion of abstinent smokers reduced by half to 5% and 25% 2. Intervention coverage to 70% 3. Including full training cost	1. Magnitude of differences remained 2. Gains decrease but results are similar 3. Same cost saving potential, to the same health gains (QALY 0.02 vs. 0.03)
** Digital or telephone counseling**			
Feenstra	One-Way sensitivity analyses	1. Cessation rate varied by their 95% CI 2. Intervention costs between 3. Discount rate for cost and outcomes: 0%, 3% and 5% 4. Discount rate cost: 4% and outcomes 0% 5. Offered to: 10 to 50% of smokers Time horizon 20, 30 and 50 years	Minimal counseling is cost-saving All other interventions are in the north-east quadrant of the CE-plane but show cost-effectiveness under EUR 25,000/QALY
Rasmussen	One-Way sensitivity analysis combined with a multivariate sensitivity analysis	1. Reducing the LYS reduced by half 2. Reducing quit rate – 6% points to 50% 3. Increasing cost by 20% Variation of discount rate for LYS between 0 and 5% p.a.	All ICER cost-effective under EUR 25,000/LYS. Range between 10.48/LYS (0% discount rate) and EUR 399.17/LYS (5% discount rate)
Smit	Deterministic sensitivity analysis: 1,000 repetitions of CEA with just completed cases	n.a.	Approx. 30% probability to be cost-effective under EUR 25,000/QALY (MTC); approx. 100% under EUR 35,000/QALY; approx. 18% to be cost-effective under EUR 25,000/QALY (MT)
Stanczyk	Deterministic sensitivity analysis: 1,000 repetitions of CEA with just completed cases	1. Using seven-day Point-prevalence-abstinence 2. Exclude surgery cost 3. Exclude respondent and productivity cost 4. Exclude medication cost	5. Reduce probability of cost-effectiveness for virtual Counseling to 50% and increase probability of telephone counseling to be cost-effective 2. Similar result than baseline 3. Similar result than baseline 4. Decreased probability for cost-effectiveness for telephone counseling and virtual counseling. Overall: Approx. 40% probability of cost effectiveness under EUR 25,000/QALY irrespective for VC and 20% for TC.
Tomson	One-Way sensitivity analysis	1. Reducing life years lost from 8 years to 6, 4, and 2 years. 2. Reducing rate of quitters from 30% to 25,20,15,10,7, and 6% 3. Discount rate cost and outcomes = 0, 3 and 5% 4. Life expectancy between 72 and 80 years	Approx. 100% probability of cost-effectiveness under EUR 25,000/LYS
Trapero-Bertran	Probabilistic sensitivity analysis	Transition probabilities and utilities = Beta distribution Relative risks and odds ratios = log-normal distribution Utility decrements due smoking-related diseases = normal distribution Costs = gamma distribution	96% of ICER replication in south-east quadrant of the CE-plane; approx. 100% probability of cost-effectiveness under EUR 25,000/QALY
Wu 2013	Probabilistic sensitivity analysis	PSA based on probability distribution based on Briggs et al., 2006	Approx. 57% Probability for cost-effectiveness under EUR 25,000/QALY (6 month and lifetime)
** Reimbursement**			
Cadier	Probabilistic sensitivity analysis	Cessation rate = log-normal distribution Cost and participation rate = triangular distribution Number and frequency of attempts, discount and inflation rate = discrete distribution	Approx. 100% probability of cost-effectiveness under EUR 25,000/LYS
Chevreaul	Probabilistic sensitivity analysis	Outcomes and cost = Continuous distribution number and frequency of attempts, discount rate, inflation rate = discrete	Approx. 100% probability of cost-effectiveness under EUR 25,000/LYS
Gebreslassie	Probabilistic sensitivity analysis	n.a.	98% of ICER replication in south-east quadrant of the CE-plane (societal perspective); approx. 99% probability of cost-effectiveness under EUR 25,000/QALY
Kaper	One-Way sensitivity analysis	Relapse rate between 20 and 50%	Approx. 95% probability for cost-effectiveness under EUR 25,000/QALY irrespective of relapse rate.
Vemer	One-Way Sensitivity analysis	Discount rate: 0 and 4%; Literature based abstinence rate (+ 2.7% pt) and additional users (+ 1.6%) incl. the variation of their 95% CI	Approx.100% probability of cost-effectiveness under EUR 25,000/QALY
** Intervention for Awareness Building**			
Gilbert	Deterministic sensitivity analysis: 1,000 repetitions of CEA with just completed cases	n.a.	Approx. 24% probability of cost-effectiveness under EUR 25,000/QALY (6 month); approx. 86% probability of cost-effectiveness under EUR 25,000/QALY (lifetime)
Kotz	One-Way sensitivity analysis	Proportion of abstinent smokers reduced by half (0.035% rather than 0.07%)	Doubling the ICER, but still cost-effective under EUR 25,000/LYS
Németh	Probabilistic sensitivity analysis	Cost = gamma distribution QoL = beta distribution Relative risk = log-normal distribution	Approx. 60% probability of cost-effectiveness under EUR 25,000/QALY
Wu 2018	Deterministic sensitivity analysis: 1,000 repetitions of CEA with just completed cases	n.a.	Approx. 83% probability of for cost-effectiveness under EUR 25,000 EUR/QALY (lifetime); approx. 26% probability of for cost-effectiveness under EUR 25,000/QALY (6-month)

*CI* Confidence interval, *n.a.* not applicable, *PSA* Probabilistic sensitivity analysis, *QoL* Quality of Life

Variations in one-way sensitivity analyses reflect similar cost and benefit differences, frequently validating ICER estimates across different scenarios. Notably, many sensitivity analyses indicate almost 100% cost-effectiveness when applying the EUR 25,000/QALY or EUR 25,000/LYS threshold. However, the results demonstrate that program intensity, the perspective of the CEA, and the time horizon can significantly affect outcomes. For example, Nohlert et al. found that high-intensity training had an 80% probability of being cost-effective, low-intensive training had only a 20% probability [40]. Gebreslassie et al. confirmed robust results of cost savings from a societal perspective, with 98% of replications falling within the south-eastern quadrant of the CE-plane [33]. Gilbert et al. illustrated that the cost-effectiveness probability of awareness building measures is only 24% for a six month time framework, but increases to 83% when using a lifetime perspective [34]. Also, Gómez Martínez found that 88.1% of ICER replications were in the south-eastern quadrant of the CE-plane, with a 96% probability of remaining cost-effective under the EUR 25,000/QALY threshold of [36]. Moreover, both studies reporting negative ICERs in the northwestern quadrant demonstrated robust sensitivity analyses results. Stanczyk et al. reported a 40% probability of cost-effectiveness for virtual counseling in their probabilistic sensitivity analyses and 20% for telephone counseling under the willingness-to-pay (WTP) threshold [45]. Likewise, Virtanen et al. showed consistent one-way sensitivity analysis results, confirming the inferiority of the face-to-face counseling compared to standard of care [49]. Also, the variation of quitting rates and the number of people affected by the intervention are essential drivers for cost-effectiveness. Hence, most simulations are using Markov models, changes on the transition probabilities, explained uncertainty of the models.

### Quality assessment

The quality assessment of the included studies demonstrated appropriate methodological quality of cost-effectiveness analysis [29–37, 39–51]. A summary of this assessment is provided in the appendix (A3). Only one study exhibited weak methodological quality and poor reporting of results [38]. It is worth noting that the study may not have reported the costing and outcome measures adequately, which regrettably meant that the evaluation of credibility and time adjustment could not be carried out. It appears that the sensitivity analysis was not performed in this study in accordance with the standards of CEA reporting. Also, the outcomes were not presented consistently, and the report included one ICER for a specific age group. This meant that the overall quality assessment for the study was not as strong as it could have been. Nearly all studies had limited external validity concerning European populations. This is primarily because CEAs are typically based on national healthcare programs, which may not be generalizable across countries [29–51]. All studies applied an incremental analysis of their costs and outcomes [29–51]. However, three studies lacked a clearly defined research question, which may affect the interpretability of their findings [34, 45, 46]. The HTA report by Gilbert provided more information on the intervention and its cost-effectiveness than the peer-reviewed article by Wu, despite being based on the same study [35, 51].

## Discussion

This systematic literature review analyzed the cost-effectiveness of non-pharmacological or combined smoking cessation programs from the perspective of public health providers and society. A total of 23 evaluation studies were identified, analyzing four key intervention types: face-to-face counseling, digital or telephone counseling, reimbursement of cessation therapies, or awareness-raising campaigns. The included studies differ in the methodological approach and analysis methods. Variations exist in study design, cost components, and outcome definitions. In particular, differences in applied methodology, particularly effectiveness parameters, discount rates, time horizons, and model assumptions pose challenges for a uniform assessment of cost-effectiveness and cost-benefit across all cessation programs. For the evidence synthesis, we applied the commonly used cost-effectiveness thresholds of EUR 25,000/QALY and EUR 50,000/QALY to assess the reported ICERs [26, 27, 55]. Based on this benchmark, nicotine and tobacco cessation programs with an ICER below EUR 25,000/QALY or EUR 25,000/LYS are classified as cost-effective. According to this criterion, 60 out of 69 ICERs indicate cost-effectiveness, regardless of whether QALY or LYS were used as effectiveness measure.

The current evidence underlines that smoking cessation is an effective and cost-efficient intervention that can lead to significant improvements in both quality of life and lifespan. Notably, studies that adopt a lifetime perspective in their cost-effectiveness analyses tend to assess the interventions more favorably than those that consider only the short-term intervention periods. For example, a study using a population-based model with a 10-year observation period reported that standard care dominated the counseling intervention [49]. In contrast, using a Markov model with a lifetime perspective, results suggest that the same counseling intervention would be preferable to standard care. This discrepancy underscores the importance of the time horizon in evaluating intervention outcomes. Supporting this, Stanzcyk et al. suggest that improvements in quality of life are typically perceived by study participants only after sustained abstinence over a longer period [45]. This study reinforces previous findings [52] affirming the economic and public health benefits of cessation programs. On average, the cost per person is EUR 1,578 (SD = EUR 2,177), though there is substantial variation across individual studies. Similarly, the effectiveness parameters differ widely, reflecting heterogeneity in intervention types and their respective methodologies. A comparison of intervention types reveals differences in cost-effectiveness, emphasizing the need for tailored approaches to maximize impact.

The most cost-effective programs are those that focus on raising awareness from a lifetime perspective, as they can reach a large number of people. Awareness-raising measures, such as Stop Smoking Days, are inexpensive and rated as effective. Nationwide marketing campaigns or personalized information letters, therefore, provide an important basis for cost-effective smoking cessation, as they inform and mobilize individuals motivated to quit. Digital counseling services show mixed results. Telephone counseling is rated as cost-effective by the studies and shows moderate costs, but also moderate effectiveness. The included studies assess text-based interventions (cost-) effective only when followed by personal counseling in the form of individual coaching or group therapy. This review builds on previous reviews, which found that electric-assisted nicotine cessation programs have low effectiveness and reported comparable ICERs of between EUR 3,435/QALY and EUR 5,153/QALY [14, 56, 57]. Additionally, the review supports previous evidence on the importance of a personalized approach for the effectiveness of cessation services. Fang et al. examined the effectiveness of eHealth cessation programs in a systematic review [58]. The authors demonstrate that personalized or interactive programs slightly increase the effectiveness of the intervention, as measured by the quit rate. Similarly, Lindson et al. report that counseling alongside pharmacotherapy enhances its effectiveness [59]. Chen et al. also recommend subsequent personal contact for telephone counseling [57]. The systemic review indicates that hotlines without personal follow-up (including group therapy) are less effective [57].

While face-to-face counseling interventions are more cost-intensive, they tend to be more effective. Compared to other types of intervention, face-to-face counseling programs show significantly higher additional costs. Low-intensity counseling programs can be cost-saving [40, 49]. In this study, community-based, face-to-face counseling services are rated as cost-effective and have approximately comparable additional costs [52]. While the interventions resulted in higher costs than the control groups, which often consisted of standard care or doing nothing, the additional costs were offset by the potential savings from the reduced negative health impacts of effective smoking cessation [40]. This suggests that preventive measures, implemented before nicotine-induced harm occurs, are less costly. Further, interventions for smokers willing to quit are more effective than those for individuals who have to stop smoking involuntarily [57]. Therefore, low-barrier nicotine and tobacco cessation programs are crucial, as they can reach more people willing to quit smoking and are also more effective than interventions for heavy smokers [56]. This is particularly important from an equity perspective, since smoking behavior is strongly associated with socio-economic status.

### Limitations

This systematic review has some limitations. First, the heterogeneity of the studies prevented a meta-analysis, as the number of studies to include was too small. Second, a meta-analysis of all studies was not feasible, as no standard errors were reported for the relevant parameters or studies used different variation parameters in their models. For the subsample analysis across the identified categories, the subsamples were too small for a meta-analysis to achieve reliable results. The limited number of studies in the category of awareness-building initiatives also restricts the evidence synthesis on cost-effectiveness in this field. Another issue in assessing study results stems from the lack of a universally defined standard of care. The standard of care is not consistently defined, neither across nor within countries, leading most studies to use different comparators. Furthermore, despite the comprehensive search query, relatively few studies were included in the full-text screening. While this may indicate a high level of sensitivity in the search, it could also indicate low precision. To address this potential limitation, grey literature, and protocols were also screened to minimize the risk of bias from missing relevant studies.

Lastly, we did not perform a full Risk of Bias (RoB) assessment, as there is currently no comprehensive RoB tool specifically designed for economic evaluations. However, we applied the Drummond checklist for CEA, which can be considered a partial RoB assessment and helps to appraise the validity and potential biases of the included CEAs. We see that the discounting rates vary between the studies, not only in the rate of discounting, but also within the studies for costs and effectiveness. This makes comparison even more challenging and is not in line with the current EUnetHTA guideline [60].

## Conclusion

Smoking cessation programs are widely available across many European countries, whereas pharmacological treatments are often excluded from public reimbursement schemes. While several meta-analyses confirm the cost-effectiveness of pharmacotherapy, our review identified a critical research gap regarding the cost-effectiveness of non-pharmacological approaches, particularly within European health systems.

This review demonstrates that most studies favor smoking cessation interventions over standard care. Among the identified sub-categories, face-to-face counseling (whether individual or in group settings) demonstrates the strongest evidence for cost-effectiveness, often demonstrating dominance over standard care. Reimbursement strategies for smoking cessation also increase the effectiveness of existing interventions.

Digital applications present mixed results in terms of cost-effectiveness, with studies suggesting that combining digital tools with face-to-face counseling is the most effective strategy for increasing abstinence rates in larger groups. However, a major gap remains in Eastern Europe, where smoking prevalence is high, but cost-effectiveness data is scarce. Future research should prioritize these regions to assess the economic viability of smoking cessation interventions within diverse healthcare systems.

## Supplementary Information

Below is the link to the electronic supplementary material.

Supplementary Material 1 (DOCX 49.7 KB)

## Data Availability

This is a systematic literature review and all data will be reported in the manuscript and could be used by other researchers.

## Footnotes

1 For calculating INB we used the formular INB = EUR 25,000/QALY*net-outcome – net-cost and for ICER = incremental cost – incremental effectiveness. Based on the current estimated Willingness-to-Pay (WTP) thresholds for Europe, we used the common societal perspective thresholds of EUR 25,000/QALY and EUR 50,000/QALY [26, 27].
